# TCMI-F-6D benchmark construction and quantitative assessment of interdisciplinary foundational competencies in traditional Chinese medicine informatics using large language models

**DOI:** 10.3389/frai.2026.1780967

**Published:** 2026-04-07

**Authors:** Zhaohang Teng, Jing Chang, Yongxiang Xu, Hongxing Kan, Yangting Ou, Jili Hu, Zongyun Gu, Yinfeng Yang, Jianhua Shu

**Affiliations:** School of Medical Information Engineering, Anhui University of Chinese Medicine, Hefei, China

**Keywords:** cognitive hierarchy theory, disciplinary knowledge structure theory, interdisciplinary competency assessment, large language models, TCMI-F-6D, traditional Chinese medicine informatics

## Abstract

**Introduction:**

Traditional Chinese Medicine Informatics (TCMI), as an emerging interdisciplinary field, places high demands on foundational interdisciplinary competency assessment in its talent cultivation and research practices. However, Large Language Models (LLMs) currently lack a suitable quantitative assessment system tailored to the characteristics of TCMI.

**Methods:**

To address this gap, this study, grounded in Cognitive Hierarchy Theory and Disciplinary Knowledge Structure Theory, selected six core disciplines closely related to TCMI from the Massive Multitask Language Understanding (MMLU) dataset, constructed an evaluation framework for foundational interdisciplinary competency in TCMI-related scenarios, and established the TCMI-F-6D (the TCMI-Foundation-6 Domain Benchmark) together with a composite metric system. Three experiments were conducted to evaluate the models’ baseline capability, learning gains, and performance stability. The experiments comprehensively assessed the competency of 20 LLMs across 8 categories, and selected 6 models with weaker overall performance for focused analysis of their interdisciplinary competency characteristics.

**Results:**

The results showed that, among the base models, ChatGLM3-6B performed best in interdisciplinary knowledge integration (43.97%), while DeepSeek-V3.1 achieved the best overall application performance (80.87%) among the chat models. Specifically, Qwen-14B-Chat also demonstrated stable and predictable learning performance under varying example conditions, with an average learning gain of 5.60% and a 95% confidence interval (CI) of [5.50%, 5.70%].

**Discussion:**

Collectively, this study clarifies the differences in foundational interdisciplinary competency among LLMs in this discipline, providing a quantifiable assessment framework, methodological support, and empirical evidence for TCMI’s educational, research tool selection, and the implementation of a standardized interdisciplinary competency assessment system.

## 1 Introduction

Traditional Chinese Medicine Informatics (TCMI) is an emerging second-level discipline that integrates traditional Chinese medicine and information science. Its highly interdisciplinary nature places strict requirements on practitioners in terms of the acquisition, transformation, dissemination, and utilization of TCM information (Cui et al., 2009). Although a multi-level talent training system covering undergraduate and master’s degrees has been established in this field since it was listed as a key second-level discipline by the State Administration of Traditional Chinese Medicine in 2009, there is still a lack of a targeted evaluation system and quantitative method to determine whether mainstream Large Language Models (LLMs) can effectively adapt to their interdisciplinary application needs.

Existing studies have demonstrated substantial progress in both the interdisciplinary evaluation and medical applications of large language models (LLMs). With respect to interdisciplinary evaluation, benchmark datasets have evolved from MMLU (Hendrycks et al., 2020), which initially assessed broad knowledge coverage, to subsequent efforts addressing data contamination through MMLU-CF (Zhao et al., 2025), emphasizing advanced reasoning capabilities via MMLU-Pro (Wang et al., 2024), and filling the gap in Chinese-language evaluation with CMMLU (Li et al., 2024), thereby establishing a more refined, fair, and comprehensive evaluation framework. In parallel, LLMs have facilitated interdisciplinary collaboration in areas such as computational biology (Ke and Ribeiro, 2025) and environmental science (Ji et al., 2025), contributing to advances in scientific research, disciplinary development, and workforce training. Regarding medical applications, LLMs have achieved near-expert performance in closed-domain medical question answering (Liu et al., 2024), demonstrated the potential to support transformative human–AI collaborative paradigms in healthcare (Qiu et al., 2023), and enhanced unified visual understanding and generation in medical multimodal tasks (Lin et al., 2025). Nevertheless, despite these advances, current research has not yet developed evaluation benchmarks or quantitative metrics specifically designed to accommodate the interdisciplinary characteristics of TCMI.

To address this research gap, this study develops an interdisciplinary foundational competency evaluation framework for TCMI scenarios based on Cognitive Hierarchy Theory and Disciplinary Knowledge Structure Theory, and further establishes the TCMI Foundation-6 Domain Benchmark (TCMI-F-6D Benchmark). This benchmark selects six core interdisciplinary disciplines closely related to TCMI from the MMLU dataset to evaluate the interdisciplinary foundational competency of LLMs in traditional Chinese medicine informatics scenarios. This study focuses on the interdisciplinary foundational competency of models in TCMI scenarios, rather than conducting a systematic evaluation of the professional knowledge system of traditional Chinese medicine.

To address the limitations of traditional LLMs evaluation, which relies on a single metric and cannot comprehensively reflect models’ knowledge mastery, disciplinary balance, and in-context learning performance, this study designs a composite metric system consisting of TCMI-F-6D-Score, Mean Accuracy, CV, 1-CV, and $\Delta_{\text{peak}}$ . On this basis, three experiments are conducted to examine the models’ foundational competence under a fixed 5-shot setting, their learning gains from 0-shot to 5-shot settings, and the statistical characteristics of the average learning gain, 95% confidence interval (CI), and R^2^ across 10 groups of randomized example experiments, respectively. This provides a methodological foundation and empirical evidence for the comparison, selection, and optimization of different types of LLMs in TCMI scenarios.

The results show that different types of LLMs exhibit significant differences in different dimensions of their interdisciplinary basic capabilities. Experiment 1 shows that ChatGLM3-6B has strong knowledge integration capabilities in the Base Model, while DeepSeek-V3.1 demonstrates superior overall performance in the Chat Model. Experiments 2 and 3 further indicate that Qwen-14B-Chat has a relative advantage in example learning ability and long-term stability. The “Interdisciplinary Basic Competency Assessment Framework for TCMI Scenarios” proposed in this paper is a systematic assessment framework based on Cognitive Hierarchy Theory and Disciplinary Knowledge Structure Theory. It focuses on the interdisciplinary basic competence of LLMs in the TCMI scenario, and is formed from subject selection, benchmark construction, and indicator design.

In conclusion, the core contributions of this study are reflected in three aspects: First, an interdisciplinary foundational competency evaluation framework is established for TCMI scenarios, expanding the conventional evaluation paradigm that relies predominantly on single tasks or isolated metrics. Second, a comprehensive metric system is proposed to systematically characterize knowledge mastery, interdisciplinary balance, and learning gain profiles, thereby providing a multidimensional perspective on model performance. Third, this study establishes the TCMI-F-6D, an interdisciplinary foundational competency benchmark tailored to TCMI scenarios, namely the TCMI-F-6D, providing a methodological foundation and empirical evidence for the comparison, selection, and optimization of relevant LLMs.

## 2 Dataset and task configuration

### 2.1 Data sources and disciplinary mapping

**Figure 1:**
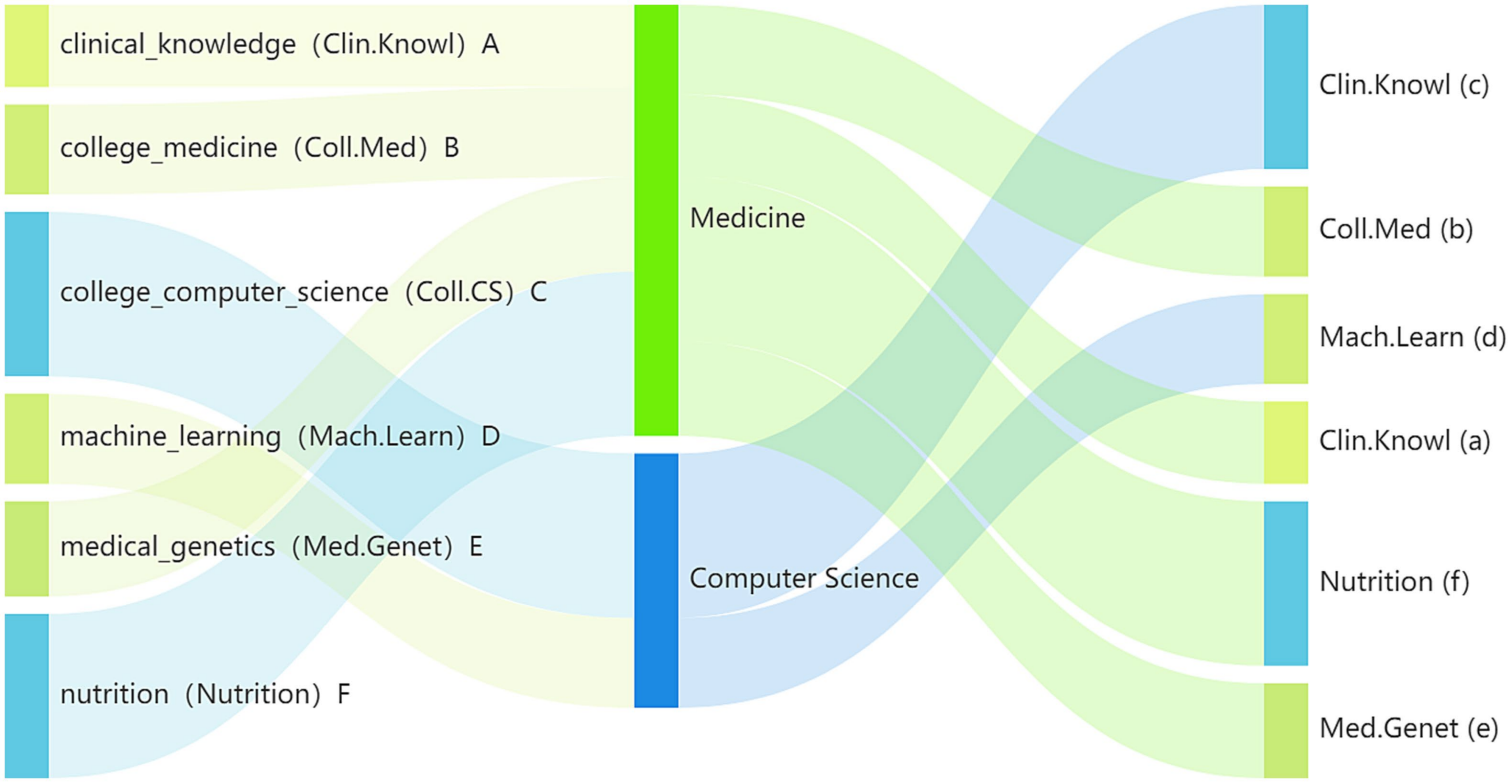
Hierarchical mapping relationship of six related disciplines.

To evaluate the domain-specific capabilities of the models in TCMI, we selected six highly relevant subjects from the MMLU dataset, abbreviated as Clin.Knowl, Coll.Med, Coll.CS, Mach.Learn, Med.Genet, and Nutrition. These subjects fall into two overarching disciplinary categories—Medicine and Computer Science—and their hierarchical correspondence to the TCMI curriculum, along with the underlying “discipline–category–course” structure, is illustrated in Figure 1.

### 2.2 Theoretical foundations

This study constructs TCMI-F-6D based on Cognitive Hierarchy Theory and Disciplinary Knowledge Structure Theory. The Cognitive Hierarchy Theory adopts the revised Bloom’s Taxonomy of Educational Objectives (Anderson and Krathwohl, 2001), while the Disciplinary Knowledge Structure Theory integrates Bruner’s concept of subject structure (Bruner, 2009) and Klein’s framework of interdisciplinary integration (Klein, 2010).

#### 2.2.1 Cognitive hierarchy theory

The Revised Bloom’s Taxonomy (Anderson and Krathwohl, 2001) has been widely adopted as a framework for assessing the development of higher-order thinking skills, including memory, comprehension, application, analysis, evaluation, and creation (Nehru et al., 2025), and has been further extended to the assessment of AI-related educational competencies (Chen et al., 2024). However, the emergence of generative AI has disrupted the traditional linear cognitive hierarchy, giving rise to a nonlinear interactive network that challenges the linear assumptions underlying this theoretical framework (Jain and Samuel, 2025). In practice, this disruption has manifested as an imbalance in cognitive skill development. Empirical evidence indicates that both large language models themselves (Jere, 2025) and students in AI-assisted learning environments (Hui, 2025) exhibit pronounced weaknesses at the level of “application.”

In response to these challenges, academic research has primarily focused on three dimensions: the assessment environment, assessment subjects, and cognitive frameworks. Specifically, researchers have explored AI-driven virtual simulation platforms to create more realistic assessment scenarios (Nehru et al., 2025), shifted the assessment focus from learners to evaluating LLMs as educators (Chen et al., 2024), and redefined human–machine collaboration in the evaluation and creation stages within reconstructed cognitive frameworks (Jain and Samuel, 2025).

In conclusion, the cognitive assessment reconstruction strategies proposed in the literature to address the challenges posed by LLMs may help enhance the interdisciplinary core competencies of TCMI professionals in the era of LLMs.

#### 2.2.2 Disciplinary knowledge structure theory

The Disciplinary Knowledge Structure Theory is grounded in Bruner’s structuralist educational thought, which emphasizes the intrinsic structure of disciplines (Bruner, 2009). Building on this perspective, Mao et al. systematically elucidated the internal relationships among core disciplinary structures, spiral curricula, and discovery learning (Mao et al., 2024). Empirical evidence further suggests that this theoretical framework can enhance students’ implicit understanding in specific instructional contexts, such as English language teaching (Wen, 2025). At the interdisciplinary level, Klein conceptualized the evolution of disciplines from juxtaposition to integration (Klein, 2010), while Pimentel et al. proposed a hierarchical framework for characterizing different levels of interdisciplinarity (Pimentel and Cho, 2022). In addition, recent empirical research by Theis and Light (2024) has provided further evidence supporting the applicability of these interdisciplinary principles in educational practice.

In conclusion, this theoretical framework provides conceptual support and methodological guidance for cultivating interdisciplinary competence in TCMI based on LLMs by internalizing TCM theory through spiral curricula, integrating information technology within interdisciplinary frameworks, and enhancing the perception of tacit knowledge through structured instruction.

### 2.3 Assessment benchmarks

Based on the theories discussed above, the development of TCMI-F-6D is grounded in two core dimensions: knowledge structure design and cognitive level assessment. First, within the knowledge structure dimension, domain adaptation was performed using MMLU. Following the principle of “Integration of Medicine and Engineering,” we selected six core disciplines from a pool of 57 subjects to establish the benchmark assessment content (Table 1), aimed at evaluating the model’s knowledge capacity in the interdisciplinary domain of traditional medicine and modern information technology. Second, within the cognitive level dimension, we classified the questions into four progressive levels based on the Revised Bloom’s Taxonomy of Educational Objectives, to assess the model’s higher-order cognitive abilities, ranging from recall to analysis.

(1) Memory: recall of basic concepts and facts (example: Which of the following is the commonest cause of dementia in the UK?).
(2) Comprehension: interpretation of principles and distinctions between concepts (example: Which of the following is NOT a symptom of anaphylaxis?).
(3) Application: transfer of knowledge and problem-solving (Example: If a gas occupies 0.1 L at 200 atm, what will its volume be at 1 atm?).
(4) Analysis: breakdown of complex information and identification of relationships (example: Given a Neural Net with N input nodes, no hidden layers, one output node, with Entropy Loss and Sigmoid Activation Functions, which of the following algorithms?).

**Table 1:** Interdisciplinary knowledge structure of TCMI-F-6D based on medicine and engineering.

Module	Domain	Assessment objective
Basic Medicine	Clin.Knowl	Assess clinical reasoning ability and verify its alignment with the logic of TCM Syndrome Differentiation and Treatment.
	Coll.Med	Evaluate fundamental medical mechanisms to consolidate the basis for constructing interdisciplinary medical knowledge graphs.
Cross-application	Nutrition	Examine the knowledge reserves regarding dietary intervention and disease prevention, based on the theory of “Food and Medicine Homology.”
	Med.Genet	Evaluate molecular biology knowledge to support micro-level research in precision medicine and traditional Chinese medicine pharmacology.
Technical method	Coll.CS	Investigate algorithmic and system principles to verify the computational capability for processing multimodal TCM data.
	Mach.Learn	Assess core Machine Learning algorithms to examine the technical potential for building intelligent TCM applications.

### 2.4 Assessment metrics

This study employs a multidimensional evaluation framework to systematically measure the knowledge mastery, interdisciplinary balance, and learning gain profiles of LLMs’ capability within the domain of TCMI. Specifically, knowledge mastery and interdisciplinary balance represent the static foundational performance of the models, while learning gain characterizes their dynamic improvement capability under few-shot conditions. Collectively, these three dimensions provide a comprehensive portrait of model performance. Building upon this, a composite metric defined as the TCMI-F-6D-Score is constructed to provide a generalized representation of the overall model performance. Detailed descriptions of the metrics and their respective directions are presented in Table 2.

**Table 2:** Experimental sources and performance thresholds of the core evaluation metrics.

Indicator name	Evaluation dimension	Positive threshold	Experiment
TCMI-F-6D-Score	Overall capability	Greater than or equal to the median of all models	1
Mean accuracy	Knowledge mastery		
1-CV	Balance term		
CV	Interdisciplinary balance	Less than or equal to the median of all models	
$\Delta {\mathrm{Acc}}_{\text{peak}}$	Learning gain	Data complete and value greater than 0	2,3
$\Delta {\mathrm{Acc}}_{\text{peak}\_\text{mean}}$	Average learning gain	Data complete and value greater than 0	3
95% CI	Stability	Narrower intervals	
R^2^	Predictability	Close to 1	

#### 2.4.1 Knowledge mastery

This metric is defined as the arithmetic mean of the 5-shot accuracies across the six core disciplines and is used to quantify the breadth of the measure its overall mastery of TCMI-related foundational knowledge under a unified 5-shot setting. The rationale for using 5-shot accuracy as the observational indicator is that, in this study, the 5-shot setting can more stably reflect the model’s ability to retrieve relevant knowledge and answer questions with limited exemplar guidance. Meanwhile, the dynamic improvement from the 0-shot condition to the shot setting at which the highest accuracy is achieved is captured by the subsequent $\Delta_{\text{peak}}$ metric. Knowledge mastery can be formulated as follows:


$$\bar{\mathrm{Acc}}=\frac{1}{6}\sum_{i=1}^{6}{\mathrm{Acc}}_{i(5-\text{shot})}$$

where $\Delta_{i(5-\text{shot})}$ denotes the 5-shot accuracy of the model on the i -th core discipline, and $\bar{\mathrm{Acc}}$ represents the average 5-shot accuracy across the six core disciplines.

#### 2.4.2 Interdisciplinary balance

The coefficient of variation (CV) of accuracies across disciplines is used to evaluate the balance of model performance. A lower CV indicates the absence of pronounced disciplinary weaknesses and reflects stability across domains, which is essential for tasks requiring integrated, multi-disciplinary knowledge application.


$$\mathrm{CV}=\frac{\sigma }{\bar{\mathrm{Acc}}},\text{where}\;\sigma =\sqrt{\frac{1}{6}\sum_{i=1}^{6}{\left({\mathrm{Acc}}_{i(5-\text{shot})}-\mathrm{Acc}\right)}^{2}}$$

where $\Delta_{i(5-\text{shot})}$ denotes the 5-shot accuracy of the model on discipline i , and $\bar{\mathrm{Acc}}$ denotes the mean 5-shot accuracy across the six disciplines. The CV is used to characterize the dispersion of model performance across disciplines. A smaller CV indicates less variation in performance across disciplines and a more balanced distribution of capabilities, whereas a larger CV suggests greater disparities in performance across domains and may imply the existence of disciplinary weaknesses. To maintain directional consistency among all evaluation metrics, 1 − CV is further adopted as a positively oriented transformation of balance, such that a larger value indicates more stable performance across disciplines.

#### 2.4.3 Learning gain

Defined as the performance gain from the zero-shot baseline to the peak accuracy, this metric quantifies the model’s capacity for knowledge transfer and adaptive learning in interdisciplinary contexts. It reflects the extent to which the model improves after being provided with the optimal number of examples. If zero-shot data are unavailable, the metric is assigned a value of 0.

In addition to static knowledge performance, the model’s adaptive learning ability under limited example prompting constitutes an important dimension of its foundational interdisciplinary competency in TCMI. To quantify the improvement from the 0-shot baseline to the highest accuracy attained across the 1-shot to 5-shot settings, this study defines the learning gain metric, $\Delta {\mathrm{Acc}}_{\text{peak}}$ , as follows:


$$\Delta {\mathrm{Acc}}_{\text{peak}}=\max({\mathrm{Acc}}_{1},{\mathrm{Acc}}_{2},{\mathrm{Acc}}_{3},{\mathrm{Acc}}_{4},{\mathrm{Acc}}_{5})-{\mathrm{Acc}}_{0}$$

where ${\mathrm{Acc}}_{n}$ denotes the model’s accuracy under the n -shot setting, ${\mathrm{Acc}}_{0}$ denotes the 0-shot accuracy, and ${\mathrm{Acc}}_{1}$ to ${\mathrm{Acc}}_{5}\;$ correspond to the accuracies under the 1-shot to 5-shot settings, respectively. This metric reflects the maximum improvement in accuracy that the model can achieve with limited example support, and can be used to characterize its ability to utilize prompt information, transfer knowledge, and adapt to the task. If 0-shot data are unavailable, $\Delta {\mathrm{Acc}}_{\text{peak}}$ is set to 0.

In this study, model learning performance from 0-shot to 5-shot is evaluated under both a fixed-example condition and multiple random-example conditions. The fixed-example condition is used to examine model performance under a consistent prompting setting, whereas the multiple random-example conditions are used to assess the stability of $\Delta {\mathrm{Acc}}_{\text{peak}}$ and the variability of results across different random example combinations.

#### 2.4.4 Average learning gain

It should be further noted that, under multiple random example conditions, the mean learning gain ( $\Delta {\mathrm{Acc}}_{\text{peak}\_\text{mean}}$ ) is introduced as a supplementary metric based on the learning gain $\Delta {\mathrm{Acc}}_{\text{peak}}$ obtained under each random example condition. Specifically, for each random example experiment, the learning gain is defined as the highest accuracy attained across the 1-shot to 5-shot settings minus the 0-shot accuracy, and is denoted as $\Delta {\mathrm{Acc}}_{\text{peak}}$ for that experiment. The $\Delta {\mathrm{Acc}}_{\text{peak}}$ values obtained from all K random example experiments are then averaged to yield the mean learning gain, $\Delta {\mathrm{Acc}}_{\text{peak}\_\text{mean}}$ , which is defined as follows:


$$\Delta {\mathrm{Acc}}_{\text{peak}\_\text{mean}}=\frac{1}{K}\sum_{i=1}^{K}\Delta {{\mathrm{Acc}}_{\text{peak}}}^{\left(i\right)}$$

where K denotes the number of random example experiments, and $\Delta {{\mathrm{Acc}}_{\text{peak}}}^{\left(i\right)}$ denotes the learning gain obtained in the i -th random example experiment. In Experiment 3, a total of 10 random example experiments were conducted. Therefore, $\Delta {\mathrm{Acc}}_{\text{peak}\_\text{mean}}$ reflects the average gain from the 0-shot baseline to the best performance attained within the 1-shot to 5-shot settings across these 10 random example conditions.

Relationship and Difference Between $\Delta {\mathrm{Acc}}_{\text{peak}}$ and $\Delta {\mathrm{Acc}}_{\text{peak}\_\text{mean}.}$

$\Delta {\mathrm{Acc}}_{\text{peak}\_\text{mean}}$ is not a new definition of learning gain; rather, it is the mean summary of $\Delta {\mathrm{Acc}}_{\text{peak}}$ across multiple random-example experiments. Specifically, $\Delta {\mathrm{Acc}}_{\text{peak}}$ denotes the maximum accuracy improvement, relative to the 0-shot baseline, achieved by a model from 1-shot to 5-shot under a given example set, whereas $\Delta {\mathrm{Acc}}_{\text{peak}\_\text{mean}}$ reflects the average level and stability of this improvement across different example selections. Its calculation follows a “compute within each group first, then average across groups” procedure, and therefore differs from the result obtained by “averaging accuracy by shot first, then computing the peak gain.”

#### 2.4.5 Overall capability

To establish a unified evaluation standard, the TCMI-F-6D Score is proposed, combining the three dimensions of knowledge mastery, interdisciplinary balance, and learning gains. This metric provides a comprehensive reflection of both the model’s static knowledge base and its dynamic learning potential.


$$\text{TCMI}-F-6D-\text{Score}=\bar{\mathrm{Acc}}\times (1-\mathrm{CV})+\Delta {\mathrm{Acc}}_{\text{peak}}\times w$$

In this formulation, $\bar{\mathrm{Acc}}\times (1-\mathrm{CV})$ represents the baseline performance term, reflecting the breadth of the model’s knowledge coverage and its cross-disciplinary balance, whereas $\Delta {\mathrm{Acc}}_{\text{peak}}$ is defined as the learning gain term to further characterize the model’s dynamic potential under few-shot conditions. Since both $\bar{\mathrm{Acc}}$ and $\Delta {\mathrm{Acc}}_{\text{peak}}$ are derived from accuracy increments and thus admit a consistent numerical interpretation, they can be jointly incorporated into the composite evaluation framework under appropriate weighting.

In this study, the weight assigned to the learning gain term is denoted by w. To mitigate the subjectivity of weight assignment, a sensitivity analysis was performed to examine how the composite score varies with different values of w. The results show that when w = 0.1, 0.2, and 0.3, the Relative Impact of the learning gain term on the composite indicator is approximately 2.57, 5.14, and 7.71%, respectively. Specifically, when w = 0.2, the AdditionVal across models falls within the interval [−6.16, 5.74], corresponding to a Range Width of 11.90. Taking the Baseline Typical Value of 0.2316 as the reference, the Relative Impact is calculated as $(11.90\times 10^{-3})÷0.2316\approx 5.14\%.$

From the results, when w = 0.1, the distinguishing effect of the learning gain term is relatively limited, making it difficult to fully reflect the dynamic differences of the model under cue-based learning conditions; while when w = 0.3, the impact of the learning gain term on the comprehensive results is relatively enhanced, which may lead to a relative weakening of the weight of the basic performance term in the overall evaluation. Considering stability, interpretability, and the relative balance between the learning gain term and the basic performance term, this paper finally adopts w = 0.2 as the default setting (Supplementary Table 1).

It is important to emphasize that this paper does not consider w = 0.2 as the only optimal parameter, but rather as an empirical setting adopted in conjunction with the results of sensitivity analysis. Its purpose is to allow the learning gain term to play a moderate auxiliary role in the comprehensive score. Therefore, the TCMI-F-6D-Score is more suitable as an interpretable comprehensive evaluation indicator to assist in comparing the comprehensive ability level of models in the TCMI basic cross-task, rather than replacing the independent analysis of each indicator.

In addition, to further characterize model performance under conditions with randomly selected examples, Experiment 3 introduces two statistical indicators, namely the 95% CI and the goodness of linear fit (R^2^). The 95% CI is used to describe the interval estimate range of $\Delta {\mathrm{Acc}}_{\text{peak}\_\text{mean}}$ , thereby reflecting the variability of the model’s average learning gain and assessing its stability. The R^2^ is used to measure the goodness of fit of the trend in model learning performance, thereby reflecting the predictability of that trend.

## 3 Experiment

### 3.1 Experimental setup and task design

This study constructs an evaluation framework based on the MMLU dataset to systematically evaluate the interdisciplinary foundational competencies of LLMs in TCMI scenarios. The TCMI-F-6D is intended to characterize the interdisciplinary foundational competencies required to support TCMI applications, rather than to correspond directly to the professional knowledge modules of traditional Chinese medicine. All experiments were conducted on a system equipped with seven GPUs (24 GB VRAM each) using PyTorch 2.6.0, CUDA 12.4, and Python 3.11. The input–output length was constrained to 1,000 tokens to ensure full coverage of the problem statements.

**Figure 2:**
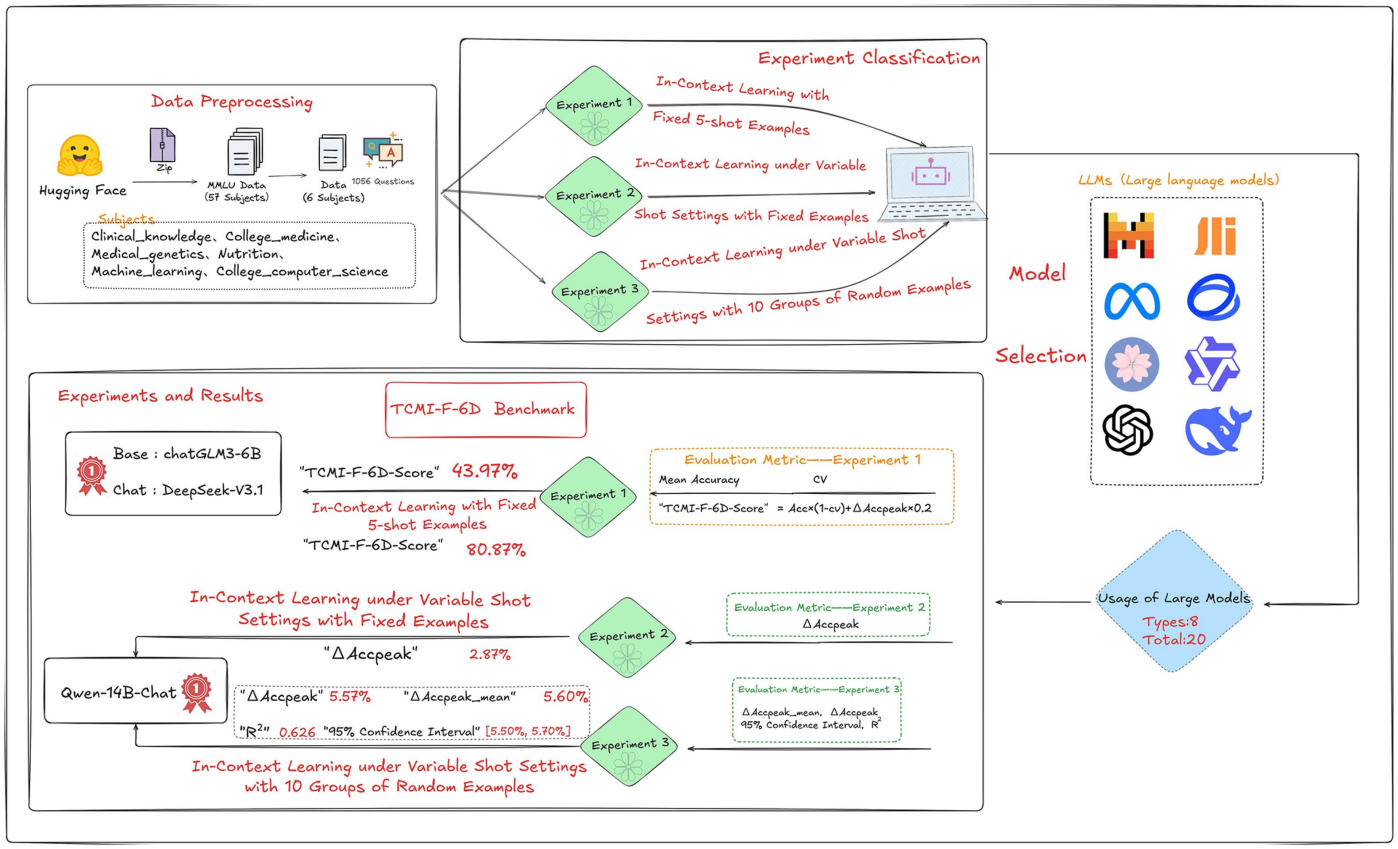
Task pipeline of the TCMI benchmark experiments.

A total of three experiments were designed in this study to evaluate the models from three perspectives, namely interdisciplinary foundational competencies, learning gain, and stability. The TCMI-F-6D-Score was used only for the comprehensive evaluation in Experiment 1. Experiment 2 was designed to analyze performance variation and learning-gain characteristics under different numbers of exemplars. Experiment 3, based on a variable-shot in-context learning setting with 10 groups of randomly sampled exemplars, was further conducted to evaluate learning gain, mean learning gain, stability, and predictability. The results of each experiment were interpreted in light of the definitions and applicable scopes of the corresponding metrics (see Figure 2 for the task pipeline).

#### 3.1.1 Experiment 1: in-context learning with fixed 5-shot examples

The first five questions of each of the six selected subjects were used as fixed exemplars. A uniform 5-shot evaluation was then performed. This experiment was designed to examine the models’ baseline performance across these six subjects, analyze their knowledge mastery and interdisciplinary balance, and accordingly calculate the composite TCMI-F-6D score.

#### 3.1.2 Experiment 2: in-context learning under variable shot settings with fixed examples

Five questions from each of the six subjects were randomly sampled to construct a fixed exemplar set, on which 0- to 5-shot evaluations were conducted. By holding exemplar content constant to eliminate interference caused by content differences, this controlled-variable design enables a systematic analysis of performance trends as the number of shots increases, thereby evaluating the model’s in-context learning capability and learning gain.

#### 3.1.3 Experiment 3: in-context learning under variable shot settings with 10 groups of random examples

This experiment was based on 10 groups of random examples and conducted evaluations from 0-shot to 5-shot across the six subjects. It focused on analyzing fluctuations in the models’ learning performance, the stability of learning gains, and trend predictability under different example conditions to examine the stability of their in-context learning performance.

#### 3.1.4 Data and baseline models

The experimental dataset comprises six subjects—Clin.Knowl, Coll.Med, Med.Genet, Mach.Learn, Coll.CS, and Nutrition—with a total of 1,056 questions. Twenty representative LLMs were selected for systematic evaluation, covering diverse parameter scales, architectures, and regional backgrounds. These include the ChatGPT series (GPT-4-Turbo, GPT-3.5-Turbo), the Mistral series (7B-v0.1, 7B-v0.3), the LLaMA series (Llama-2-13B, Llama-2-7B-hf), Bloom-7b1, the Baichuan2 series (7B-Base, 13B-Base, 13B-Chat), the ChatGLM series (2-6B, 3-6B, 4.5, 4-9B-chat-hf), the Qwen series (2-7B, 3-14B-Base, 14B-Chat), and the DeepSeek series (LLM-7B-Base, R1-Distill-Qwen-14B-Chat, V3.1).

Experiment 1 evaluates all 20 models. Based on the results of Experiment 1, Experiment 2 selected the six models that generally lower TCMI-F-6D-Score and $\bar{\mathrm{Acc}}$ values and conducted stepwise evaluations from 0-shot to 5-shot to examine their learning gain characteristics. Experiment 3 further employed the same six models as those used in Experiment 2 and performed in-context learning evaluations under variable shot settings with 10 groups of random examples to analyze their mean learning gain, performance fluctuations, and trend characteristics under different example conditions.

## 4 Results and discussion

This study aims to examine the relationship between LLM categories (Base Models and Chat Models) and their interdisciplinary foundational competencies. Twenty LLMs are classified and evaluated by comparing their accuracy, mean performance, and other relevant metrics across six academic disciplines. In the experimental design, Experiment 1 adopts a fixed 5-shot prompting setting and is primarily intended to examine the models’ baseline performance. Since $\Delta {\mathrm{Acc}}_{\text{peak}}$ reflects the maximum increase in accuracy achieved as the number of examples increases relative to the 0-shot baseline; this metric is more suitable for evaluating the learning gain characteristics of the models in Experiment 2. Meanwhile, Experiment 3 was conducted in-context learning evaluations under variable shot settings with 10 groups of random examples, and further introduces $\Delta {\mathrm{Acc}}_{\text{peak}\_\text{mean}}$ , 95% CI, and R^2^ to analyze the models’ mean learning gain, range of fluctuations, and trend predictability under different example conditions.

Question 1: Which Base-type LLM is most suitable for serving as a comprehensive subject teacher?

Answer 1: ChatGLM3-6B.

Under identical base model conditions, the performance of LLMs on cross-disciplinary tasks is summarized in Table 3. The results show that ChatGLM3-6B delivers the strongest overall performance across most disciplines. Although its accuracy in the Nutrition domain is slightly lower than that of Qwen2-7B, it achieves an overall mean accuracy of 48.72%. The next-best performer is Qwen3-14B, which exhibits particularly strong results in the Machine Learning domain.

**Table 3:** Evaluation accuracies of base models.

Model	Clin.knowl	Coll.med	Coll.CS	Mach.learn	Med.genet	Nutrition	Average
Bloom-7b1	26.79	29.48	26.00	28.57	36.00	22.55	28.23
Llama-2-7b-hf	26.79	15.03	28.00	25.89	32.00	25.82	25.59
Mistral-7B-v0.3	24.53	24.28	30.00	21.43	30.00	20.92	25.19
Mistral-7B-v0.1	24.91	15.61	26.00	25.89	30.00	23.20	24.27
Qwen3-14B	44.91	42.20	47.00	**41.96**	43.00	50.33	44.90
Baichuan2-13B	29.43	26.01	20.00	31.25	20.00	22.88	24.93
DeepSeek-LLM-7B	19.62	22.54	31.00	29.46	27.00	27.78	26.23
Qwen2-7B	36.60	41.62	38.00	27.68	40.00	**52.61**	39.42
ChatGLM3-6B	**50.19**	**49.71**	**51.00**	38.39	**53.00**	50.00	*48.72*
ChatGLM2-6B	45.28	39.88	39.00	40.18	42.00	48.04	42.40
Baichuan2-7B	20.75	20.23	23.00	27.68	21.00	27.12	23.30

Bold values in columns 2-7 denote the maximum value in each column, while bold italic values in the Average column denote the maximum average value.

**Figure 3:**
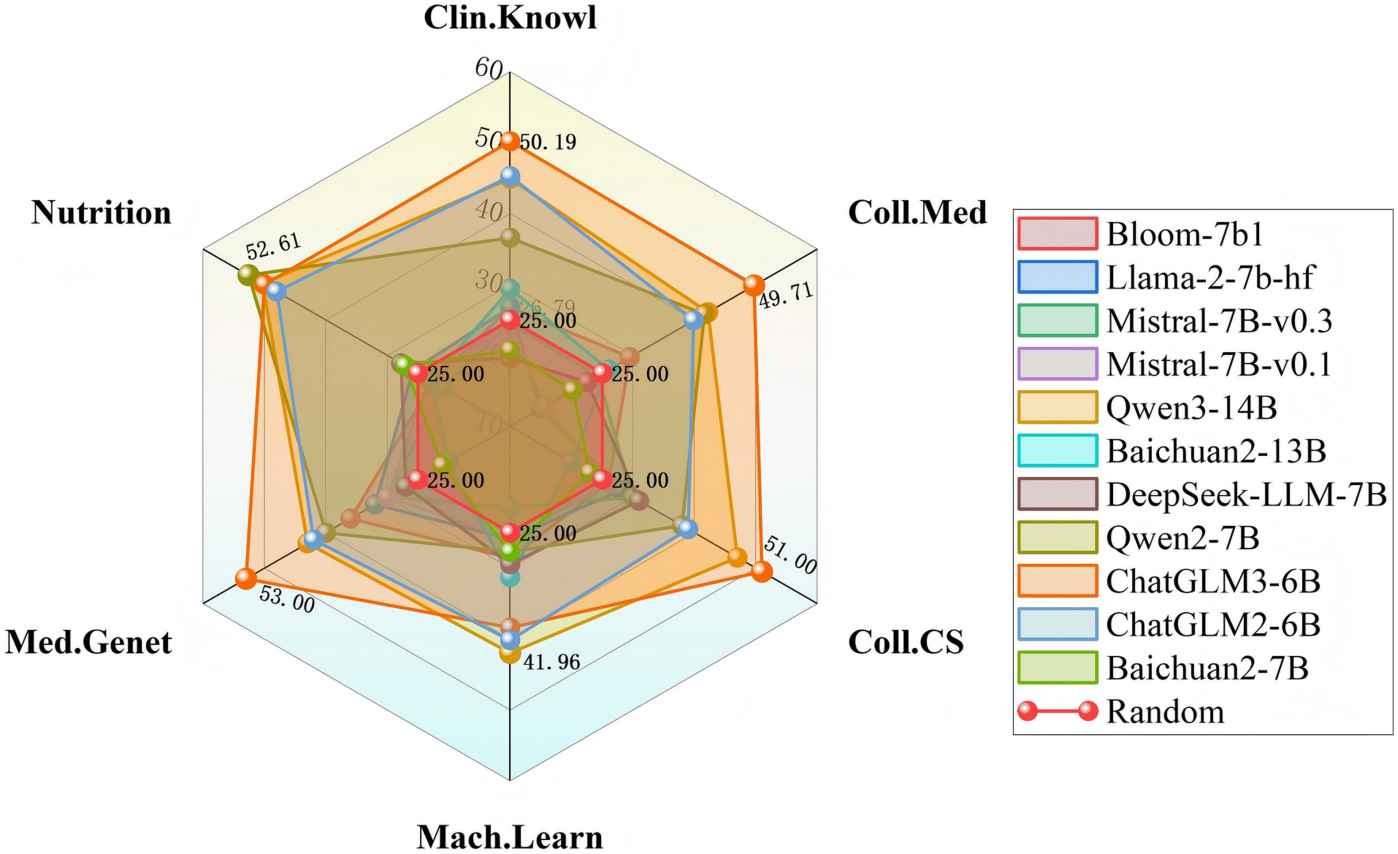
Performance profiles of domestic and international base models across six disciplines.

In contrast, models such as Baichuan2-7B, Mistral-7B-v0.1, and Baichuan2-13B demonstrate relatively low accuracy across multiple disciplines, with notably weak performance in specialized domains such as Coll.Med and Clin.Knowl. These findings indicate that their capacity to comprehend complex interdisciplinary content and generate high-quality responses requires further improvement. Peak performance for each discipline is illustrated in Figure 3.

Question 2: Which Chat-type LLM is most suitable for serving as a comprehensive subject teacher?

Answer 2: DeepSeek-V3.1.

Under identical chat model conditions, LLMs exhibit differences in performance on interdisciplinary tasks, as shown in Table 4. DeepSeek-V3.1 and ChatGPT-4-turbo form the top-performing tier, achieving average accuracies of 85.60 and 83.27%, respectively. DeepSeek-V3.1 led in Coll.CS and Mach.Learn, whereas ChatGPT-4-turbo showed advantages in Clin.Knowl and Coll.Med. Notably, both models achieved an outstanding accuracy of 94.00% in Med.Genet, demonstrating comparable excellence in this particularly challenging discipline.

**Table 4:** Evaluation accuracies of Chat models.

Model	Clin.knowl	Coll.med	Coll.CS	Mach.learn	Med.genet	Nutrition	Average
ChatGPT-4-turbo	**87.55**	**86.71**	74.00	71.43	**94.00**	85.95	83.27
ChatGPT-3.5-turbo	67.17	61.27	41.00	42.86	65.00	63.40	56.78
Llama-2-13B	22.64	23.12	26.00	31.25	25.00	21.24	24.88
DeepSeek-V3.1	85.66	84.97	**85.00**	**77.68**	**94.00**	86.27	*85.60*
ChatGLM4.5	82.64	72.83	69.00	75.89	80.00	**87.91**	78.05
ChatGLM-4-9B-Chat-hf	58.11	55.49	44.00	41.07	62.00	56.86	52.92
Qwen-14B-Chat	35.85	42.77	36.00	29.46	37.00	39.22	36.72
DeepSeek-R1-Distill-Qwen-14B	31.32	26.59	30.00	34.82	33.00	25.82	30.26
Baichuan2-13B-Chat	48.38	36.99	30.00	29.46	42.00	50.65	39.63

Bold values in columns 2-7 denote the maximum value in each column, while bold italic values in the Average column denote the maximum average value.

**Figure 4:**
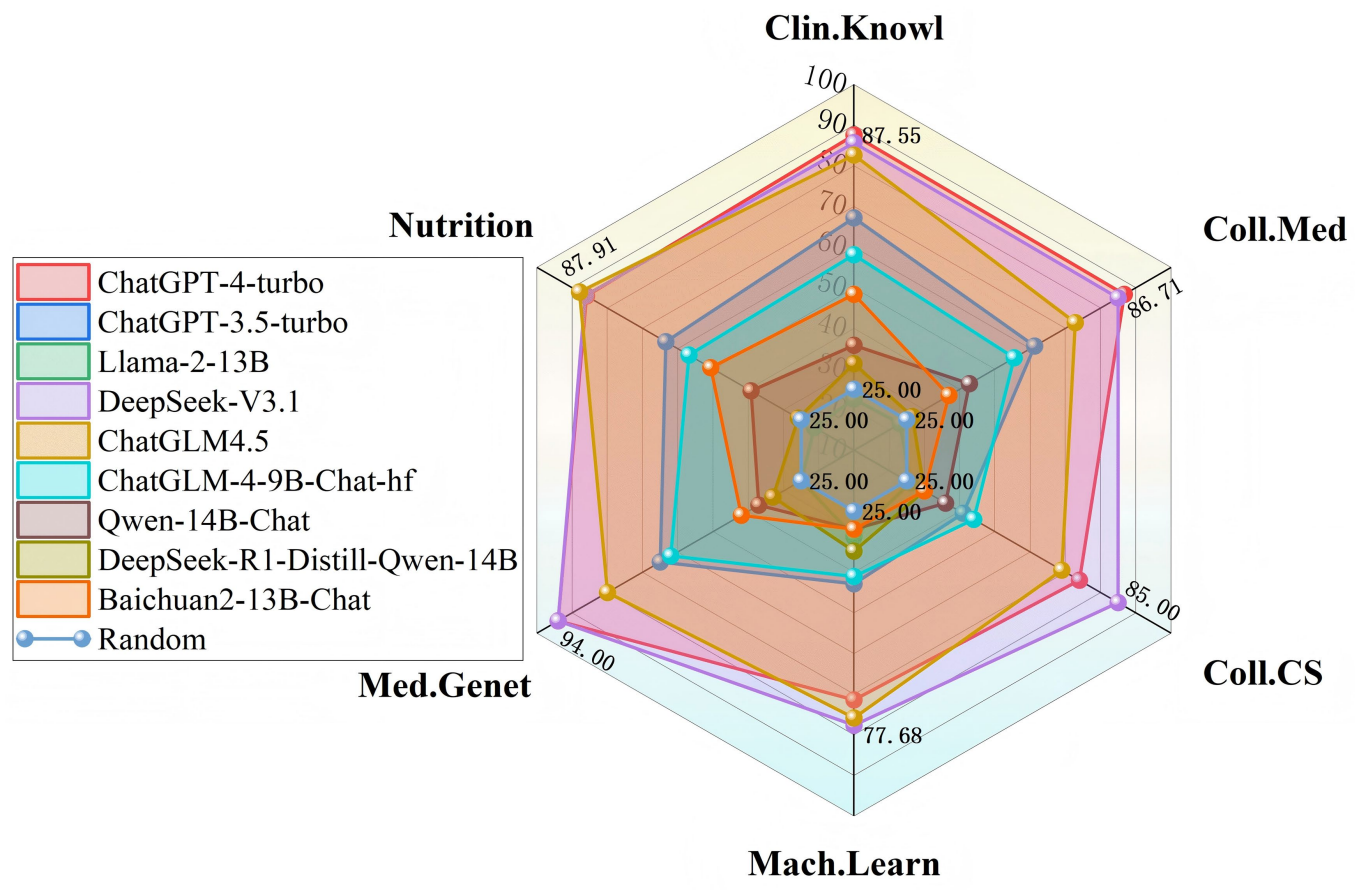
Performance profiles of domestic and international Chat models across six disciplines.

Following closely is ChatGLM4.5, which achieves an average accuracy of 78.05%, notably attaining the highest score among all models in the Nutrition domain (87.91%), indicating its distinct advantage in this specific field. In contrast, ChatGPT-3.5-turbo and ChatGLM-4-9B-Chat-hf exhibit substantially lower performance, with average accuracies of only 56.78 and 52.92%, respectively, suggesting potential limitations when handling complex cross-disciplinary content. The peak performance in each discipline is illustrated in Figure 4.

Question 3: Which Base model demonstrates the strongest overall capability without instruction fine-tuning?

Answer 3: ChatGLM3-6B.

Experiment 1, which conducted a comprehensive evaluation of multiple open-source base models (see Table 5), shows that ChatGLM3-6B significantly outperforms other models in both mean accuracy (48.72%) and overall capability score (43.97%), indicating that it achieves the highest level of knowledge mastery. Qwen3-14B also performs well, ranking second in overall capability score (41.91%). Notably, its CV is only 6.65%, reflecting the highest stability (1–6.65% = 93.35%) and indicating highly consistent performance across different disciplines.

**Table 5:** Evaluation metrics of base models (Experiment 1).

Model	Mean accuracy	Coefficient of variation	TCMI-F-6D-Score
Bloom-7b1	28.23	14.56	24.12
Llama-2-7b-hf	25.59	20.18	20.43
Mistral-7B-v0.3	25.19	14.49	21.54
Mistral-7B-v0.1	24.27	18.04	19.89
Qwen3-14B	44.90	**6.65**	41.91
Baichuan2-13B	24.93	17.50	20.57
DeepSeek-LLM-7B	26.23	15.05	22.28
Qwen2-7B	39.42	18.73	32.04
ChatGLM3-6B	**48.72**	9.74	**43.97**
ChatGLM2-6B	42.40	7.65	39.16
Baichuan2-7B	23.30	13.01	20.27
Median-Baseline	26.23	14.56	22.28

Bold values denote the optimal value in each column. For Mean Accuracy, TCMI-F-6D-Score, and Accuracy, the bold value indicates the maximum value, whereas for Coefficient of Variation, the bold value indicates the minimum value.

In contrast, some models, such as Llama-2-7b-hf, exhibit the highest CV (20.18%), indicating substantial variability across disciplines, and its overall score (20.43%) falls below the median baseline value (22.28%). Among the models, Qwen3-14B achieves the best CV (6.65%), followed by ChatGLM2-6B (7.65%) and ChatGLM3-6B (9.74%), suggesting that both models demonstrate relatively strong and consistent performance across disciplines.

Regarding overall capability, ChatGLM3-6B achieves the highest score (43.97%). However, as shown in Table 4, its performance in Mach.Learn (38.39%) is substantially below its mean accuracy (48.72%) and differs markedly from its performance in Coll.CS (51.00%), another computer science–related discipline. This pronounced intra-domain variability highlights the model’s limitations in specific domains, particularly within the computer science field.

Question 4: Which Chat model demonstrates the strongest overall capability without instruction fine-tuning?

Answer 4: DeepSeek-V3.1.

Experiment 1, which conducted a comprehensive evaluation of multiple popular Chat models (Table 6), revealed substantial differences in both performance and interdisciplinary balance, highlighting a capability profile distinct from that of Base models. DeepSeek-V3.1 and ChatGPT-4-turbo constitute the top-performing tier; however, DeepSeek-V3.1 outperforms ChatGPT-4-turbo across multiple evaluation metrics. It achieves the lowest CV (5.53%) and the highest mean accuracy (85.60%), indicating superior interdisciplinary balance and knowledge mastery. Its composite score of 80.87% ranks first among all models.

**Table 6:** Evaluation metrics of Chat models (Experiment 1).

Model	Mean accuracy	Coefficient of variation	TCMI-F-6D-Score
ChatGPT-4-turbo	83.27	9.54	75.33
ChatGPT-3.5-turbo	56.78	18.78	46.12
Llama-2-13B	24.88	13.05	21.63
DeepSeek-V3.1	**85.60**	**5.53**	**80**.**87**
ChatGLM4.5	78.05	8.03	71.78
ChatGLM-4-9B-Chat-hf	52.92	14.46	45.27
Qwen-14B-Chat	36.72	10.95	32.70
DeepSeek-R1-Distill-Qwen-14B	30.26	10.68	27.03
Baichuan2-13B-Chat	39.58	20.80	31.35
Median-Baseline	52.92	10.95	45.27

Bold values denote the optimal value in each column. For Mean Accuracy, TCMI-F-6D-Score, and Accuracy, the bold value indicates the maximum value, whereas for Coefficient of Variation, the bold value indicates the minimum value.

ChatGLM4.5, with the second-lowest CV (8.03%) and the third-highest mean accuracy (78.05%), forms the second tier. A significant performance gap separates this tier from the third tier, represented by ChatGPT-3.5-turbo and ChatGLM-4-9B-Chat-hf, which attain composite scores of only 46.12 and 45.27%, respectively. Moreover, some models, such as Baichuan2-13B-Chat, although not at the bottom in terms of composite capability (31.35%), exhibit the highest CV (20.80%), corresponding to the lowest stability performance index (1–20.80% = 79.20%). This indicates substantial performance fluctuations across disciplines, in stark contrast to DeepSeek-V3.1 (5.53%). Additionally, Baichuan2-13B-Chat’s composite score (31.35%) falls below the median baseline (45.27%).

Although DeepSeek-V3.1 demonstrates the highest overall capability, its performance across disciplines is not uniform. As shown in Table 5, its performance in Mach.Learn and Coll.CS (77.68 and 85.00%, respectively) is below its mean accuracy (85.60%), revealing a relative “weakness” in computer science–related disciplines. This pronounced intra-disciplinary variability suggests that even advanced models do not exhibit uniform improvement across all domains, providing novel perspectives and challenges for future research on model capability boundaries, particularly in the computer science field.

Question 5: Based on the distribution of correlation strength in the heatmaps, what structural differences are observed between Base and Chat models?

Answer 5: Remarkable differences.

**Figure 5:**
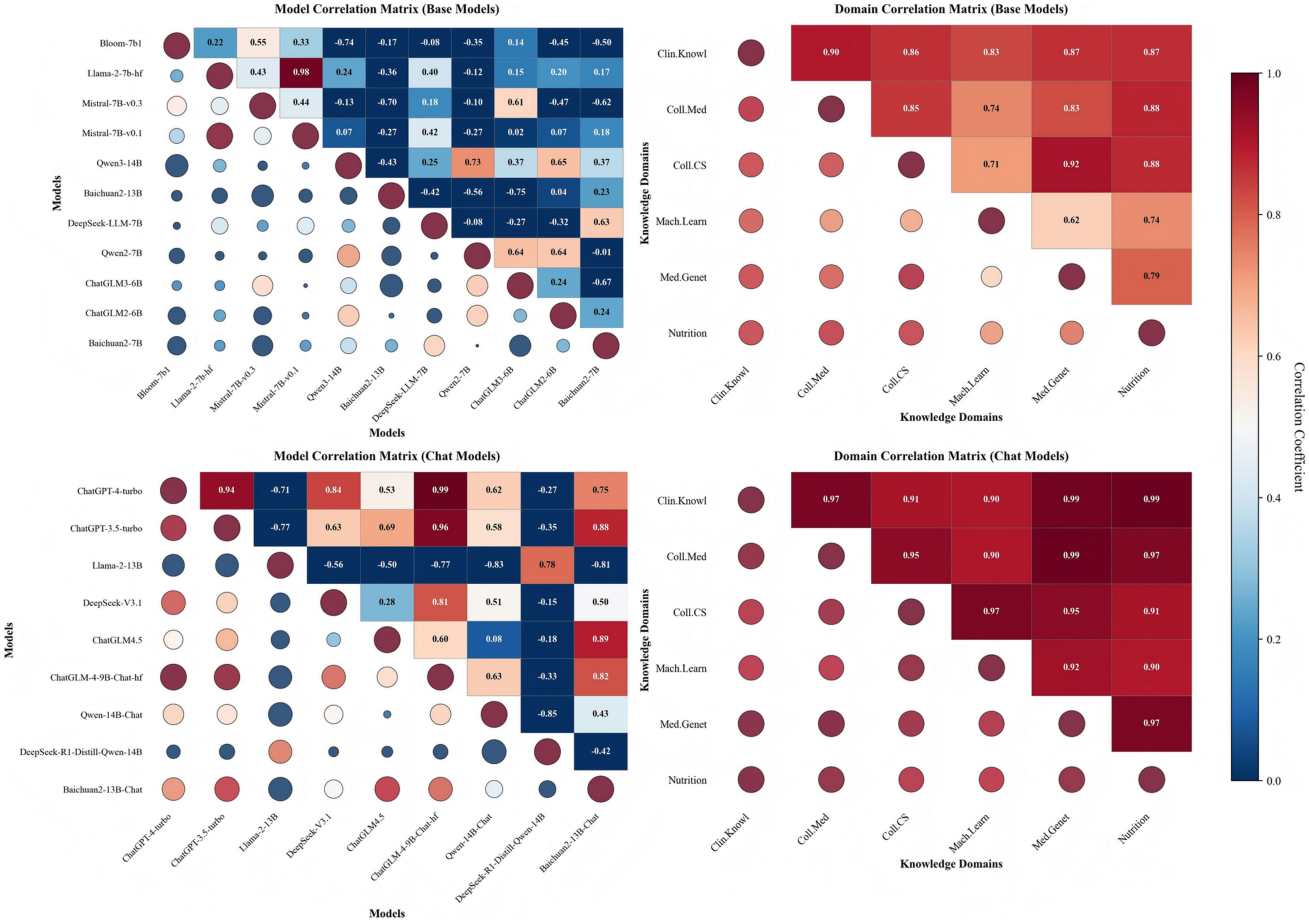
Analysis of model–domain performance correlations.

As shown in Figure 5, the correlation matrix of Base models exhibits a “multi-centred” distribution, with substantial variation in inter-model correlations. For instance, Llama-2-7b-h and Mistral-7B-v0.1 show a strong positive correlation (r = +0.98), whereas Qwen3-14B and Bloom-7b1 exhibit a significant negative correlation (r = −0.74). The standard deviation of all correlation coefficients is only 0.42, suggesting an initial trend toward differentiation.

In contrast, Chat models display a more pronounced “two-tier differentiation” pattern. ChatGPT-4-turbo and ChatGLM-4-9B-Chat-hf demonstrate an extremely strong positive correlation (r = +0.99), forming a highly consistent performance cluster, while Qwen-14B-Chat and DeepSeek-R1-Distill-Qwen-14B exhibit a significant negative correlation (r = −0.85), representing opposing performance tiers. The standard deviation of all correlation coefficients reaches 0.63, markedly higher than that of Base models (0.42), indicating that Chat models not only exhibit stronger performance but also superior overall capability compared with Base models.

Question 6: What clustering patterns are observed in the inter-model correlations?

Answer 6: Clear family-based clusters coexist with hybrid patterns.

The correlation heatmap (Figure 5) reveals a differentiated pattern reflecting the models’ technical lineages, mainly forming three performance clusters. The first cluster comprises the GPT family (ChatGPT-4-turbo and ChatGPT-3.5-turbo), exhibiting high internal consistency (r = +0.94). The second cluster includes Chinese language models (Qwen3-14B, ChatGLM3-6B, and ChatGLM2-6B) with an average correlation of 0.42, indicating moderate inter-model association. The third cluster consists of open-source Base models (Bloom-7b1, Llama-2-7b-hf, Mistral-7B-v0.1/v0.3), which display substantial internal variability, with inter-model correlations ranging from weak (r1 = +0.22) to strong (r2 = +0.98).

Notably, DeepSeek-V3.1 exhibits a unique hybrid correlation profile. It shows strong correlations with top-performing English Chat models (e.g., ChatGPT-4-turbo, r = +0.84) and certain Chinese models (e.g., ChatGLM-4-9B-Chat-hf, r = +0.81), but is markedly differentiated from other models and even negatively correlated with some (e.g., DeepSeek-R1-Distill-Qwen-14B, r = −0.15).

Question 7: From the perspective of knowledge integration, what structural differences are observed in cross-disciplinary associations between Base and Chat models?

Answer 7: Modular versus networked structures.

The discipline-specific heatmaps (Figure 5) clearly reveal significant structural differences in cross-disciplinary knowledge integration between Base and Chat models. Base models exhibit a modular structure, whereas Chat models display a networked structure. Specifically, the knowledge correlations within Base models are highly heterogeneous: the correlation between Coll.CS and Med.Genet reaches a maximum of 0.92, while the correlation between Mach.Learn and Med.Genet is only 0.62, resulting in a pronounced range of 0.30. This substantial disparity indicates uneven knowledge integration within Base models. In contrast, Chat models show consistently high correlations across all disciplines, ranging narrowly from 0.90 to 0.99 (range = 0.09), demonstrating superior knowledge integration capabilities.

Question 8: Which large model in Table 7 demonstrates the strongest in-context learning capability and thus has the greatest potential to serve as a cross-disciplinary teacher?

**Table 7:** Interdisciplinary few-shot learning performance of six LLMs under variable shot settings with fixed examples in Experiment 2 (mean across six disciplines).

Model	Accuracy						
	0-shot	1-shot	2-shot	3-shot	4-shot	5-shot	$\Delta {\mathrm{Acc}}_{\text{peak}}(\%)$
Baichuan2-7B-Base	26.15	25.47	25.39	**26.29**	24.49	25.21	0.14
Qwen-14B-Chat	39.03	**41.93**	35.17	33.85	35.21	32.65	2.87
DeepSeek-R1-Distill-Qwen-14B	41.29	36.16	35.10	37.93	**38.21**	37.42	−3.08
Mistral-7B-v0.3	29.06	26.23	24.90	**27.56**	24.66	24.79	−1.50
Llama-2-7b-hf	26.27	24.77	23.47	23.91	23.85	**25.68**	−0.59
Bloom-7b1	23.79	**24.54**	22.92	24.30	23.69	21.41	0.75

Bold values denote the optimal value in each column. For Mean Accuracy, TCMI-F-6D-Score, and Accuracy, the bold value indicates the maximum value, whereas for Coefficient of Variation, the bold value indicates the minimum value.

Answer 8: Qwen-14B-Chat.

As shown in Table 7, substantial differences are observed in the learning gains of different LLMs under example prompting conditions, among which Qwen-14B-Chat exhibits the strongest in-context learning ability and therefore shows the greatest potential to serve as an interdisciplinary teacher. Specifically, Qwen-14B-Chat achieves a $\Delta {\mathrm{Acc}}_{\text{peak}}$ value of 2.87%, the highest among all models, indicating that it can leverage a small number more effectively to improve its performance on interdisciplinary tasks.

In contrast, Bloom-7b1 and Baichuan2-7B achieve only modest gains of 0.75 and 0.14%, respectively, suggesting that the performance improvements they derive from example prompting are relatively weak. More notably, models such as DeepSeek-R1-Distill-Qwen-14B and Mistral-7B-v0.3 exhibit negative $\Delta {\mathrm{Acc}}_{\text{peak}}$ values, with the lowest reaching −3.08%, indicating that even their best performance under the examined example conditions remains below the 0-shot baseline. These results suggest that the effects of adding examples are not consistent across models and that the inclusion of examples does not necessarily lead to performance improvement. Negative $\Delta {\mathrm{Acc}}_{\text{peak}}$ values may be related to the models’ sensitivity to prompt format and contextual organization; when overall capability is relatively limited, the added examples may fail to provide effective assistance and may instead interfere with the models’ original judgment. In addition, inconsistencies between the models’ original knowledge-based decision patterns and the provided examples may also prevent the added examples from being translated into performance gains.

Overall, Qwen-14B-Chat demonstrates stronger in-context learning ability and more pronounced positive gains under example prompting conditions, and therefore shows greater potential to serve as an interdisciplinary teacher among the six models.

**Figure 6:**
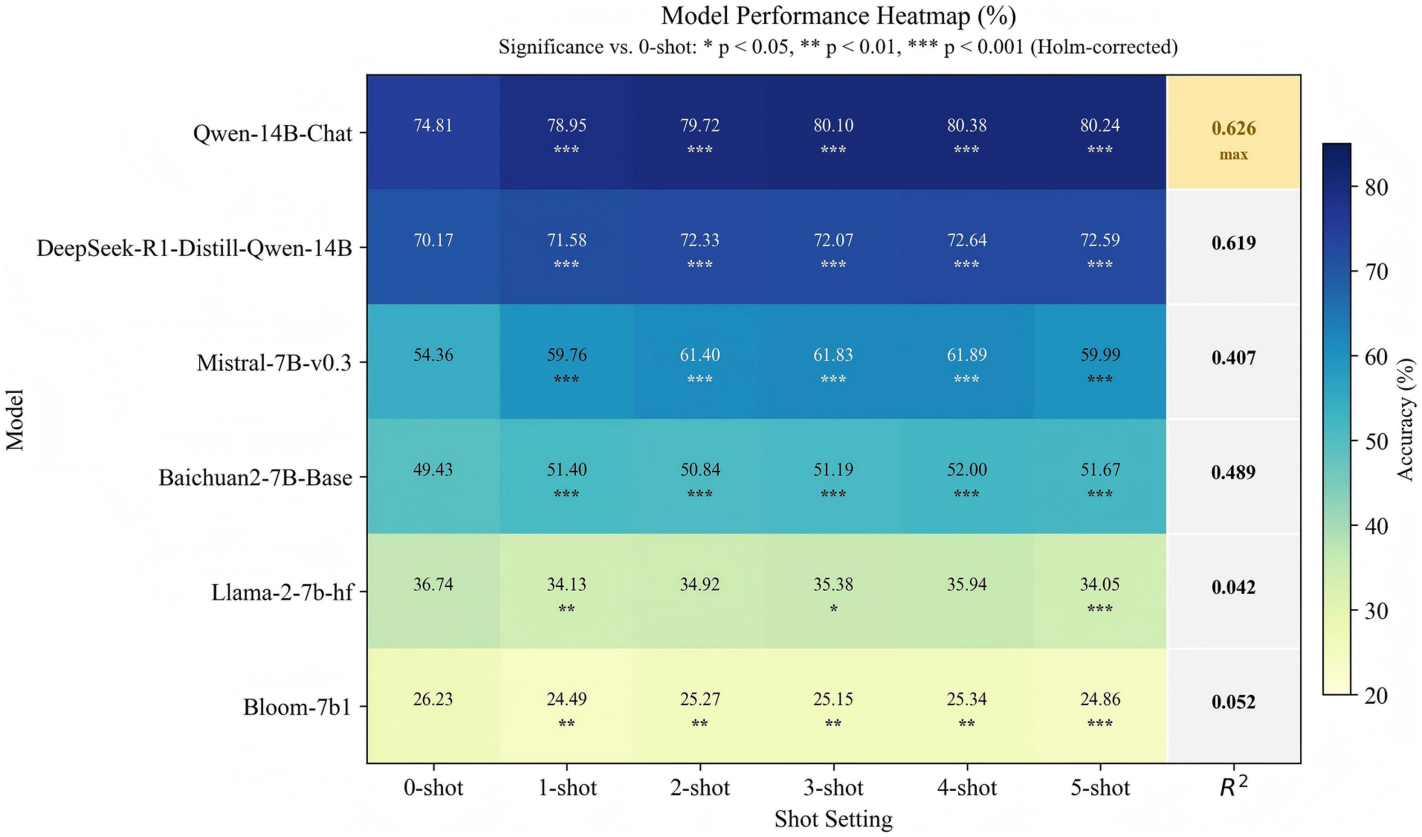
Accuracy of six LLMs from 0-shot to 5-shot and the heatmap of significance relative to 0-shot.

Question 9: Based on Table 8 and Figure 6, which large model is better suited to serve as a stable interdisciplinary teacher over the long term?

**Table 8:** Interdisciplinary few-shot learning performance of six LLMs under 10 groups of random example conditions in Experiment 3 (mean across six disciplines).

Model	Accuracy						$\Delta {\mathrm{Acc}}_{\text{peak}}(\%)$
	0-shot	1-shot	2-shot	3-shot	4-shot	5-shot	
Baichuan2-7B-Base	49.43	51.40	50.84	51.19	**52.00**	51.67	2.57
Qwen-14B-Chat	74.81	78.95	79.72	80.10	**80.38**	80.24	5.57
DeepSeek-R1-Distill-Qwen-14B	70.17	71.58	72.33	72.07	**72.64**	72.59	2.47
Mistral-7B-v0.3	54.36	59.76	61.40	61.83	**61.89**	59.99	7.53
Llama-2-7b-hf	36.74	34.13	34.92	35.38	**35.94**	34.05	−0.80
Bloom-7b1	26.23	24.49	25.27	25.15	**25.34**	24.86	−0.89

Bold values denote the optimal value in each column. For Mean Accuracy, TCMI-F-6D-Score, and Accuracy, the bold value indicates the maximum value, whereas for Coefficient of Variation, the bold value indicates the minimum value.

Based on the 10 groups of random example experiments, a further stability analysis was conducted on the few-shot learning results of each model. As shown in Table 8 and Figure 6, Qwen-14B-Chat consistently achieved the highest accuracy from 0-shot to 5-shot settings, while also exhibiting more stable learning gains and a more consistent trend of performance change. Therefore, among the six models, it appears to be the most suitable for long-term and stable application in interdisciplinary teaching.

First, in terms of accuracy, the accuracy of Qwen-14B-Chat increased from 74.81 to 80.38%. Even under the zero-shot setting, the model still demonstrated a strong ability to master interdisciplinary knowledge and significantly outperformed the other models, particularly Mistral-7B-v0.3, whose highest accuracy was only 61.89%. These results indicate that Qwen-14B-Chat can maintain strong baseline performance without relying on many examples, and that it already demonstrates strong interdisciplinary foundational competencies and question-answering ability under the zero-shot condition.

Second, in terms of the stability of learning gains, Qwen-14B-Chat achieved an average learning gain of 5.60%, with a 95% CI of [5.50, 5.70%] (Supplementary Table 2), which was the narrowest among all models. This suggests that it consistently achieved relatively uniform positive improvements under different random example conditions, demonstrating strong stability. In contrast, although Mistral-7B-v0.3 showed a higher learning gain, its 95% CI was [7.34, 8.21%], with a larger range of fluctuations, indicating relatively weaker stability in performance changes.

Third, in terms of trend predictability, Qwen-14B-Chat achieved the highest goodness of linear fit from 0-shot to 5-shot (R^2^ = 0.626), indicating that its accuracy changed more regularly and predictably as the number of shots increased. Specifically, the accuracy of Qwen-14B-Chat exhibited an overall pattern of steady increase followed by gradual stabilization in the later stages, suggesting relatively consistent performance changes across different shot settings. In contrast, the accuracy of Mistral-7B-v0.3 dropped to 59.99% at the 5-shot setting, and its R^2^ was 0.407, indicating weaker trend consistency and relatively lower overall predictability as the number of shots increased.

Finally, in terms of statistical significance, the significance heatmap in Figure 6 further supports the advantage of Qwen-14B-Chat. In the figure, “Significance vs. 0-shot” represents the significance of the differences between each setting from 1-shot to 5-shot and the 0-shot baseline, where * indicates *p* < 0.05, ** indicates *p* < 0.01, and *** indicates *p* < 0.001 (Holm-corrected). The results show that, for Qwen-14B-Chat, the accuracy improvements from 1-shot to 5-shot relative to the 0-shot baseline reached statistical significance under multiple conditions, indicating good consistency in its accuracy gains across conditions. By contrast, although Mistral-7B-v0.3 also reached statistical significance under all conditions, fewer conditions attained higher levels of significance, and its overall statistical significance was weaker than that of Qwen-14B-Chat, suggesting weaker consistency in its accuracy changes relative to the 0-shot baseline.

Considering accuracy, the stability of learning gains, trend predictability, and statistical significance together, Qwen-14B-Chat shows a greater advantage for long-term, stable applications in interdisciplinary teaching. Although Mistral-7B-v0.3 achieved the largest learning gain, its 95% CI was wider, its R^2^ was lower, and its overall statistical significance was weaker than that of Qwen-14B-Chat, indicating relatively lower result stability. Therefore, Qwen-14B-Chat is more suitable as a long-term, stable interdisciplinary teacher model.

## 5 Conclusion

This study addresses the current gap in the TCMI field, which arises from the lack of effective tools for evaluating interdisciplinary competence, by focusing on LLMs as the assessment objects. Based on Cognitive Hierarchy Theory and Disciplinary Knowledge Structure Theory, we developed the TCMI-F-6D benchmark, which encompasses a multidimensional metric system that includes the TCMI-F-6D Score, Mean Accuracy, 1-CV, CV, and $\Delta {\mathrm{Acc}}_{\text{peak}}$ , to evaluate the performance of LLMs across six core interdisciplinary domains in the MMLU dataset.

The evaluation results revealed substantial differences among the models from three perspectives: foundational capability, learning gain, and performance stability. Experiment 1, In-Context Learning with Fixed 5-shot Examples, compared the overall performance of Base Models and Chat Models. Among the Base Models, ChatGLM3-6B demonstrated relatively superior interdisciplinary knowledge integration ability (43.97%), while, among chat models, DeepSeek-V3.1 achieved the highest overall performance score (80.87%). Experiment 2, In-Context Learning under Variable Shot Settings with Fixed Examples, examined the learning gains of the models from 0-shot to 5-shot. The results showed that Qwen-14B-Chat achieved the highest learning gain (+2.87%), demonstrating the strongest example-based learning ability. Experiment 3, In-Context Learning under Variable Shot Settings with 10 Groups of Random Examples, further evaluated the stability of model learning performance under different example conditions. The results showed that Qwen-14B-Chat achieved an average learning gain of 5.60%, with a 95% CI of [5.50, 5.70%] and an R^2^ of 0.626, indicating stable and predictable accuracy improvement under varying example conditions.

The primary contributions of this study are threefold. First, it establishes an evaluation framework for foundational interdisciplinary competency tailored to the TCMI scenario, where the selection of the six core interdisciplinary subjects supported by explicit theoretical foundations rather than empirical choice from a single perspective. Second, it introduces a composite metric system consisting of the TCMI-F-6D-Score, $\bar{\mathrm{Acc}}$ , CV and $\Delta {\mathrm{Acc}}_{\text{peak}}$ metric, enabling a more comprehensive assessment of LLMs in terms of knowledge mastery, disciplinary balance, and learning gain. Third, it provides a benchmark for evaluating the foundational interdisciplinary competency of LLMs in the TCMI field, providing empirical guidance for model comparison, selection, and optimization in this domain.

Future research will focus on real-world TCMI applications and professional knowledge in traditional Chinese medicine, while extending the assessment from foundational interdisciplinary competency to practical task validation. More LLMs, including models of different types, scales, and newly released versions, and more complex task settings, will be included to further examine model performance in real-world scenarios. The proposed framework may also serve as a reference for evaluating LLMs in other interdisciplinary fields.

## Data Availability

Publicly available datasets were analyzed in this study. This data can be found here: all data used in this study are publicly available. The TCMI-F-6D benchmark was constructed from publicly available subsets of the MMLU dataset accessed via Hugging Face at: https://huggingface.co/datasets/cais/mmlu. The datasets themselves are not included in the manuscript or Supplementary materials, and no original datasets were generated in this study.
