# Supplementary material for: TCMI-F-6D benchmark construction and quantitative assessment of interdisciplinary foundational competencies in traditional Chinese medicine informatics using large language models
Source: Front Artif Intell. 2026 Apr 7;9:1780967. doi: 10.3389/frai.2026.1780967 (PMC13096100; doi:10.3389/frai.2026.1780967)
Supplement: Supplementary file 1 [file Table_1.DOCX]

Supplementary Material

# Supplementary Tables

Supplementary Table 1 Sensitivity analysis of learning capability weight

| **Model** | **AdditionVal(w×10^-3^)** | | | | $\text{∆Acc}_{\text{peak}}$ | |
| --- | --- | --- | --- | --- | --- | --- |
|  |  | 0.1 | 0.2 | 0.3 |  |  |
| Baichuan2-7B-Base |  | 0.14 | 0.28 | 0.42 |  | 1.40 |
| Qwen-14B-Chat |  | 2.87 | 5.74 | 8.61 |  | 28.70 |
| DeepSeek-R1-Distill-Qwen-14B-Chat |  | -3.08 | -6.16 | -9.24 |  | -30.80 |
| Mistral-7B-v0.3 |  | -1.50 | -3.00 | -4.50 |  | -15.00 |
| Llama-2-7b-hf |  | -0.59 | -1.18 | -1.77 |  | -5.90 |
| BLOOM-7B1 |  | 0.75 | 1.50 | 2.25 |  | 7.50 |
| Range of added value |  | [-3.08,2.87] | [-6.16,5.74] | [-9.24,8.61] |  | ----- |
| Range Width |  | 5.95 | 11.90 | 17.85 |  | ----- |
| Relative Impact（%） |  | 2.57 | 5.14 | 7.71 |  |  |

Note:

(1)AdditionVal=$\text{∆Acc}_{\text{peak}}\text{×w×}\text{10}^{\text{-3}}$；Relative Impact(%)=（$\text{Range Width×}\text{10}^{\text{-3}}$）/Baseline Typical Value。Among these, the Baseline Typical Value, which is defined as the arithmetic mean of $\overline{\text{Acc}}\text{×(1-CV)}$, is 0.2316. A weight of w=0.2 was ultimately selected because it produces an approximate 5% relative effect, a magnitude that can be characterised as “significant but non-dominant.”

(2)$\text{∆Acc}_{\text{peak}}$ is distinct from a simple accuracy difference. It specifically refers to the net gain from the baseline performance at 0-shot to the best observed performance at the maximum number of shots, thereby excluding interference caused by suboptimal choices of example quantity.

Supplementary Table 2 Mean learning gains and corresponding 95% confidence intervals for six large models across 10 randomized in-context example sets

| **Model** | ${\Delta Acc}_{peak\_mean}$ | **95% Confidence Interval** |
| --- | --- | --- |
| Mistral-7B-v0.3 | 7.77 | [7.34,8.21] |
| Qwen-14B-Chat | 5.60 | [5.50,5.70] |
| Baichuan2-7B-Base | 2.83 | [2.71,2.96] |
| DeepSeek-R1-Distill-Qwen-14B | 2.74 | [2.58,2.89] |
| Llama-2-7b-hf | 0.83 | [0.08,1.59] |
| BLOOM-7B1 | -0.35 | [-0.58,-0.12] |

Note:

${\Delta Acc}_{peak\_mean}$ denotes the mean ${\Delta Acc}_{peak}$ across 10 randomized example sets, where ${\Delta Acc}_{peak}$ represents the learning gain under a single randomized in-context example condition and is calculated as $max({Acc}_{1},{Acc}_{2},{Acc}_{3},{Acc}_{4},{Acc}_{5})-{Acc}_{0}$. The 95% confidence interval (95% CI) is calculated from these 10 randomized example sets. Notably, ${\Delta Acc}_{peak\_mean}$ is not a new definition of learning gain, but an aggregated form of ${\Delta Acc}_{peak}$ across multiple randomized example experiments; therefore, it is not numerically identical to the learning gain derived directly from shot-wise mean accuracies.
